# Food Intake and Core Body Temperature of Pups and Adults in a *db* Mouse Line Deficient in the Long Form of the Leptin Receptor without *Misty* Mutation

**DOI:** 10.1155/2018/9670871

**Published:** 2018-12-03

**Authors:** Wijang Pralampita Pulong, Miharu Ushikai, Emi Arimura, Masaharu Abe, Hiroaki Kawaguchi, Masahisa Horiuchi

**Affiliations:** ^1^Department of Hygiene and Health Promotion Medicine, Graduate School of Medical and Dental Sciences, Kagoshima University, Kagoshima City, Kagoshima 890-8544, Japan; ^2^Department of Life and Environmental Science, Kagoshima Prefectural College, Kagoshima City, Kagoshima 890-0005, Japan

## Abstract

Different involvement of leptin signaling in food intake (FI) and body temperature (BT) in pups and adults has been suggested. However, the leptin receptor (Lepr) long-form-deficient (*db*) mouse line has not been fully examined in pups. In the most available *db* mouse line, wild-type (WT) mice have a mutation in the dedicator of cytokinesis 7 gene, named *misty*, which was recently revealed to be involved in neuronal development. Therefore, we established a line of *db* mice without the *misty* mutation using natural mating. Adult (8 weeks of age) homozygous *db*/*db* mice displayed significantly higher core body weight (BW) and FI and significantly lower core BT than WT mice. However, postnatal (2 weeks of age) *db*/*db* mice displayed similar BW and milk intake and significantly lower core BT than WT mice. Correspondingly, adult and postnatal *db*/*db* mice exhibited altered mRNA levels of hypothalamic orexigenic and anorexigenic peptide in adults but not in pups. Additionally, *db*/*db* mice displayed significantly lower mRNA levels of brown adipose tissue uncoupling protein 1 at both ages. In conclusion, the *db* mouse line without the *misty* mutation clearly showed the different involvements of the Lepr long form in FI and BT in pups and adults.

## 1. Introduction

Leptin and the leptin receptor (Lepr) are important for physiological and pathological states related to body weight (BW) regulation in humans and rodents [1–5]. To date, many studies investigating leptin and Lepr have revealed important roles in energy balance, particularly food intake (FI) and body temperature (BT) regulation in adults [6, 7]. In addition, leptin and Lepr may be involved in neuronal development at infancy [8, 9]. To our knowledge, the physiological and pathological roles of leptin signaling in adults have been well characterized, but the roles of leptin signaling in pups remain to be elucidated.

To reveal the role of leptin and Lepr *in vivo*, two mutant mouse strains with ligand (*ob*/*ob* mice) or receptor deficiency (*db*/*db* mice) have been widely used as obesity and type 2 diabetes animal models, respectively [10]. In *ob*/*ob* mice, the effects of leptin administration were examined to elucidate the role of leptin in appetite and thermogenesis [6, 11]. Additionally, in *db*/*db* mice, the genetically induced rescue of Lepr was assessed to elucidate the role of leptin signaling in the hypothalamus and sympathetic nerves [12–14]. Leptin signaling is primarily mediated through orexigenic peptides, such as neuropeptide Y (NPY) and agouti-related peptide (AgRP), and anorexigenic peptides, such as proopiomelanocortin (POMC) and cocaine- and amphetamine-regulated transcript (CART), in the arcuate nucleus of the hypothalamus [1]. Specifically, leptin signaling suppresses orexigenic peptide expression and increases anorexigenic peptide expression. In addition, uncoupling proteins in brown adipose tissues (BAT) were enhanced by leptin signaling through the sympathetic nervous system [6]. However, postnatal *ob*/*ob* and *db*/*db* mice displayed similar but slightly heavier BWs to their respective controls including heterozygotes and wild-type (WT) mice [15–18]. Leptin administration experiments using WT mice revealed that leptin did not influence mRNA levels of hypothalamic orexigenic and anorexigenic peptide in pups [19]. Physiologically, similar to leptin administration, increased plasma leptin concentrations in pups is termed the leptin surge [20]. In a leptin administration study, there were different results in orexigenic and anorexigenic peptide genes' expression in pups and adults, suggesting that leptin signaling may differ between pups and adults [19, 20].

*db*/*db* mice might be useful to examine the age-dependent influence of Lepr deficiency [21]. However, *db*/*db* mice under the BKS.Cg background are generally identified by coat color because of proteins coded by the dedicator of cytokinesis 7 (*Dock7*) gene, which is near the *Lepr* gene [22, 23]. Specifically, mice with a homozygous *db* gene express the wild-type *Dock7* gene, leading to obesity and black coat color. Thus, mice with a wild-type *Lepr* gene display a homozygous mutation in the *Dock 7* gene, named *misty* (*m*/*m*), leading to a lean body type and grey coat color [24]. Recently, a Dock7 knockout mouse study indicated the Dock7 product involved in the development of neuronal cells related to the sympathetic nervous system, in addition to BW regulation [25, 26]. As a result, *m*/*m* mice have lower BWs than WT mice. Therefore, to examine the lack of Lepr signaling on BT and FI regulation in an age-dependent manner, especially in pups, a line of *db* mice without the influence of the *misty* gene should be established.

Using a *db* mouse line without the influence of *misty*, we examined FI and BT in pups and adults. In addition, the age-dependent expression of orexigenic and anorexigenic genes in the hypothalamus and uncoupling protein 1 (*Ucp1*) in the BAT was examined. Furthermore, thyroid function, which is associated with FI and BT regulation, was surveyed. In the present study, the roles of the long form of Lepr, which is specifically deficient in *db*/*db* mice, in the age-dependent regulation of FI and BT were elucidated.

## 2. Materials and Methods

### 2.1. Animals

All mice were maintained at a constant room temperature of 22 ± 2°C, with a 12 h light cycle (07:00–19:00 h) and free access to water and standard chow (14.4 kJ/g, CE-2; CLEA Japan, Tokyo, Japan). Briefly, we generated a mouse line carrying the *db* gene, a mutation in the *Lepr* gene, without a mutation in the *Dock7* gene (*misty*). First, we purchased heterozygote male mice (BKS.Cg-*Dock7^M^Lepr^db^*/*Dock7^m^Lepr^+^*; *M db/m+*) that had a black coat color and lean body type (Charles River, Kanagawa, Japan; and CLEA Japan, Tokyo, Japan). As displayed in Table 1, some mice were *M db*/*M+*, possibly because of natural recombination during breeding at the companies. A male *M db*/*M+* mouse was mated with female *M db/m+* mice, producing *M db*/*M db*, *M db/m+*, *M db*/*M+*, and *M+*/*m+*. The mating for the experiments was permitted by the company (Charles River). From the genotyping results of the pups, we obtained a pair of *Lepr* gene heterozygotes, who had the wild-type *Dock7* gene (*M db*/*M+*). Male and female *M db*/*M+* mice were mated to obtain WT (*M+*/*M+*: *+*/*+*), heterozygous (*M db*/*M+*: *db*/+), and homozygous (*M db*/*M db*: *db*/*db*) mice. We chose a colony with 5–8 litters and genotyped the mice (*+/+, db/+,* and *db/db*) at 1 week of age. Mice BWs were measured weekly from 1 to 8 weeks of age. Mice were individually recognized by black pen markings on the surface of their hand and foot. At 3 weeks of age, the mice were removed from their dams, separated by sex, and placed into a cage with littermates of the same sex. At 4 weeks of age, mice were separated and then kept individually in one cage. Male *+/+* mice and *db/db* littermates at 2 and 8 weeks of age were further examined as pups and adults, respectively. *db/db* mice at 8 weeks of age were designated as adults, although this is younger than generally defined, because older mice display a loss of hyperinsulinemic conditions [21, 22]. Mice were anesthetized with pentobarbital (100 mg/kg) after 3 h fasting to prevent influences by overfasting at infancy. Accordingly, mice at 8 weeks of age were treated after 3 h fasting. Blood was collected from the heart with a syringe containing EDTA (final concentration, 4 mM) and centrifuged at 2000 ×g for 5 min to separate plasma. Mice were then euthanized by cervical dislocation, and the brain, heart, liver, white adipose tissue (WAT, fat surrounding epididymis), and BAT were removed, blotted, weighed, and frozen immediately in liquid nitrogen. The hypothalamic block was separated from the brain [27]. The plasma and frozen organs were stored at −80°C until analysis.

All animal experiments and procedures used in the present study were approved by the Ethics Committee for Animal Experimentation at Kagoshima University (MD15004, 16035, and 16087), which is standardized to the Japanese national guidelines for animal experiments. Additionally, the principles of laboratory animal care were followed.

### 2.2. Genotyping for the Lepr and Dock7 Genes

Previously, *db*, a mutation in the *Lepr* gene, was obtained using the PCR method with mismatched primers [28, 29]. In the present study, for increased reliability, the PCR method without mismatched primers was established as follows. DNA was obtained from the tail or ear tissue from mice at 1 week of age, as described above, using the KAPA Express Extract DNA Extraction Kit (KAPA Biosystems, MA, USA). The DNA extracted was used as a template for the genes identified. The *Lepr* gene was amplified with forward and reverse primers (Supplementary Table 1) by PCR. The PCR product was digested with *Hpy*166II restriction enzyme (New England Biolabs Japan Inc.). The PCR products digested were analyzed by agarose gel (Kanto HC, Kanto Chemical Co., Inc., Tokyo, Japan) for identification of the three genotypes (*+*/*+*, *db*/*+*, and *db*/*db*). The *Dock7* gene was amplified with one forward and two reverse primers (Supplementary Table 1). The PCR products were analyzed by agarose gel, and *Dock7* genotyping (*M*/*M*, *m*/*M*, and *m*/*m*) was performed.

### 2.3. Food and Milk Intake Measurements

For adults, the weight of the diet was measured at 9:00 h. FI was then calculated as the difference in diet weight over 4 consecutive days. To measure milk intake of mice at 2 weeks of age, dams were separated from pups for 3 h. The pups were weighed 3 h after separation and again after 1 hour of suckling. Body weight gain during 1 h of suckling was utilized to estimate milk intake [30].

### 2.4. Core Body Temperature Measurements

Core BT was measured with a high traceable thermometer D642 (Tateyama Kagaku Group, Toyama, Japan). The thermocouple electrode was introduced 15 mm into the rectum of the animals.

### 2.5. Biochemical Parameters

Plasma glucose and triglyceride (TG) levels were measured with a commercial kit (Wako, Osaka, Japan). Plasma insulin, leptin, and corticosterone concentrations were measured with the respective ELISA kits (Morinaga Institute of Biological Science Inc., Kanagawa, Japan; R&D Systems, Minneapolis, MN, USA; Enzo Life Science Inc., PA, USA). Plasma hormones related to thyroid function, such as triiodothyronine (T3), thyroxine (T4), and thyroid-stimulating hormone (TSH), were measured by ELISA (Cusabio, Biotech Co., MD, USA).

### 2.6. Gene Expression

RNA from the hypothalamus and BAT was extracted using a Trizol reagent (Ambion, Carlsbad, CA, USA) following the manufacturer's instruction. Isolated RNA was treated with DNase (Ambion, Carlsbad, CA, USA) to remove genomic contamination. First-strand cDNA was synthesized using 5 *μ*g total RNA with oligo(dT)_20_ (Invitrogen, Carlsbad, CA, USA) following the manufacturer's instruction. Gene expression was determined by quantitative RT-PCR with gene-specific primers using the SYBR Green Master Mix (Roche, Mannheim, Germany) on a TAKARA detection system TP800 (TAKARA, Shiga, Japan). Relative mRNA levels were determined by the 2^-∆∆CT^ method, with *Gapdh* as a reference gene. Primer sequences are provided in Supplementary Table 1.

### 2.7. Statistical Analyses

**Figure 1:**
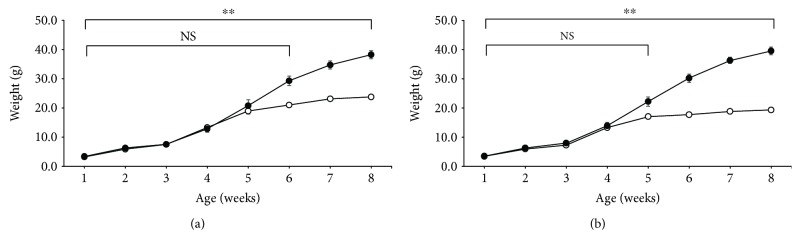
Body weight (BW) change during development. Weekly BWs of male (a) and female mice (b) are displayed. Open symbols represent WT (*+/+*) mice, and closed symbols represent *db/db* mice. The sample size of all groups is 6. The values are the mean ± SE. The data were analyzed statistically by two-way (repeated measurement) ANOVA. ^∗∗^ *P* < 0.01 compared with WT mice. NS: not significant.

**Figure 2:**
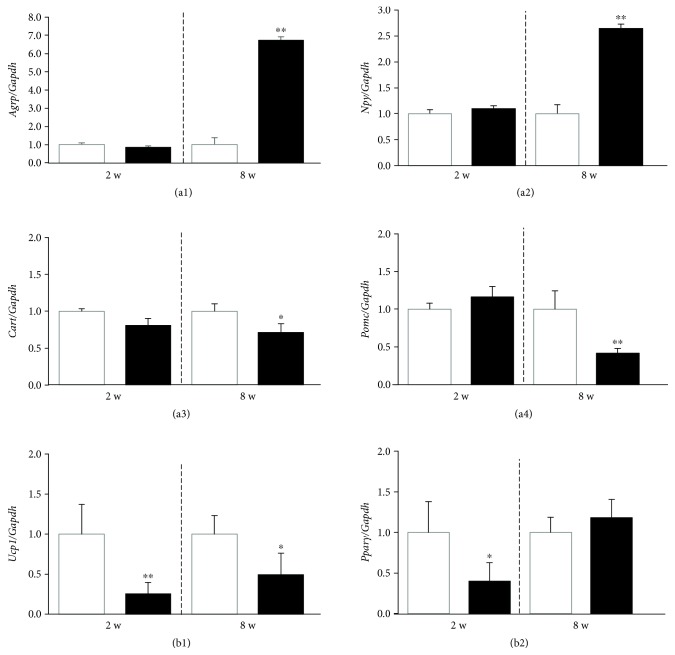
Expression of genes related to food intake and thermogenesis regulation in the hypothalamus and brown adipose tissue (BAT). Expression of genes in the hypothalamus are shown (a). *Agrp* (a1), *Npy* (a2), *Cart* (a3), and *Pomc* (a4) expression levels are displayed relative to *Gapdh* mRNA levels. Expression of genes related to thermogenesis in BAT (b). *Ucp1* (b1) and *Pparγ* (b2) mRNA levels are displayed relative to *Gapdh* mRNA levels. The sample size of all groups is 7-8. The values are the mean + SE. The values are expressed as the fold change relative to WT mice at the respective age (all values were set to 1.0). Open columns and closed columns represent WT (*+/+*) mice and *db/db* mice, respectively. The data were analyzed statistically by unpaired Student's *t*-test. ^∗^ *P* < 0.05, ^∗∗^ *P* < 0.01 compared with WT mice at the respective age.

Values are presented as the mean ± standard error (SE). Significant differences between groups in Figure 1 were identified using two-way (repeated measurement) ANOVA (SPSS, Version 23.0). Significant differences between groups in Tables 2 and 3 were identified using two-way (factorial) ANOVA, followed by the Tukey-Kramer method as a multiple comparison. For the statistical analysis in Figure 2 and in a part of Tables 2 and 3, paired and unpaired Student's *t*-tests were used as appropriate. *P* < 0.05 was considered significant.

## 3. Results

### 3.1. Establishment of a Mouse Line Carrying Lepr^db^ with Wild-Type Dock7

We classified mice with a black coat color and lean body type from the two companies as heterozygotes (expected genotype, *M db*/*m+*) (Table 1). Because of natural recombination during breeding at the companies, we obtained a male mouse carrying *db* with wild-type *Dock7* (*M db/M+*). By further mating, we then generated mice carrying *db* without the *Dock7* mutation. For the experiments, we produced mice by cross mating in the line.

### 3.2. Body Weight Differences between +/+ and db/db Mice

Figure 1 illustrates the changes in BW in wild-type mice (WT: *Lepr^+^*/*Lepr^+^*) and homozygous mutants (*db*/*db*: *Lepr^db^*/*Lepr^db^*) in male (a) and female (b) mice fed with a regular chow diet after weaning at 3 weeks of age. There were no significant differences in BW between male and female WT and *db*/*db* mice for the initial 6 and 5 weeks after birth by two-way (repeated measurement) ANOVA. Both male and female *db*/*db* mice displayed significantly higher BWs than WT mice in 7 and 6 weeks of age after birth, respectively.

### 3.3. Milk and Food Intake

As displayed in Table 2, both sexes of WT and *db/db* mice displayed a similar BW change, indicating that mice ingested a similar amount of milk during the 1 h suckling period at 2 weeks of age. Seven WT and 7 *db/db* male mice were obtained from the same four mothers. The mean litter size was 7.7 ± 1.1 and 7.9 ± 1.2, indicating no significant difference. Furthermore, 5 WT and 5 *db/db* female mice were obtained from the same three mothers and one different mother. The mean litter size was 7.6 ± 1.1 and 7.0 ± 1.0, indicating no significant difference. Therefore, the milk availability between genotypes was not influenced by the litter size. However, both sexes of *db/db* mice displayed higher FI than WT mice at 8 weeks of age.

### 3.4. Organ Weights

Organ weights, including the brain, heart, liver, WAT, and BAT, were measured in male mice (Table 2). There was a significance in the interaction between age and genotype in the brain, liver, and WAT. With a multiple comparison, the respective weight of organs such as the brain, liver, and WAT of *db/db* and WT mice was similar at 2 weeks of age and significantly different at 8 weeks of age. The heart weight was significantly different between mice at 2 and 8 weeks of age. In BAT, *db/db* mice displayed significantly higher weights than WT mice, and mice at 8 weeks of age displayed higher weights than mice at 2 weeks of age.

### 3.5. Biochemical Parameters

Only males were examined. There was a significance in the interaction between age and genotype in glucose, insulin, leptin, and corticosterone. With a multiple comparison, the values in glucose, insulin, and corticosterone of *db/db* and WT mice were similar at 2 weeks of age and significantly different at 8 weeks of age. In leptin, *db/db* mice at both ages displayed a significantly higher value than WT mice at both ages, respectively. Additionally, plasma TG levels were comparable between the two genotypes at 2 and 8 weeks of age, although mice at 8 weeks of age displayed a significantly higher value than mice at 2 weeks of age. For hormones related to thyroid function, *db*/*db* mice showed significantly lower T4 but similar T3 and TSH levels in plasma compared with WT mice.

### 3.6. Core Body Temperature

Core BT at 8:00 and 20:00 h was measured in both sexes of WT and *db*/*db* mice at 2 and 8 weeks of age (Table 3). There was not a significance in the interaction between age and genotype in BT at 8:00 and 20:00 h, respectively. Both sexes of *db*/*db* mice displayed significantly lower BTs than WT mice at 8:00 and 20:00 h, respectively. Both sexes of mice at 8 weeks of age displayed significantly higher BTs than mice at 2 weeks of age, except BT at 8:00 h of female mice between ages. Both sexes of WT and *db*/*db* mice at 8 weeks of age had significantly higher BTs at 20:00 h than those of the corresponding mice at 8:00 h. Additionally, both sexes of WT and *db/db* mice at 2 weeks of age had significantly higher BTs at 20:00 h than those of the corresponding mice at 8:00 h, except male WT mice at 2 weeks of age.

### 3.7. Expression of Genes Related to Leptin Signaling for FI and Thermogenesis Regulation

Only males were examined. To understand the mechanism underlying the role of leptin signaling in FI and thermogenesis regulation in pups and adults, we examined the mRNA levels of related genes in the hypothalamus and BAT (Figure 2). Particularly, we examined the expression of genes encoding orexigenic peptides, such as AgRP and NPY, and anorexigenic peptides, such as CART and POMC. *Agrp* and *Npy* expression levels in *db*/*db* mice at 8 weeks of age were significantly higher than those in WT mice. In contrast, *db*/*db* mice at 8 weeks of age displayed significantly lower *Cart* and *Pomc* expression in the hypothalamus compared with WT mice. Interestingly, there were no significant differences in *Agrp*, *Npy*, *Cart*, or *Pomc* expression between WT and *db*/*db* mice at 2 weeks of age. As displayed in Figure 2, *Ucp1* gene expression in BAT was significantly lower in *db*/*db* mice than in WT mice at both ages. Furthermore, expression of the gene encoding peroxisome proliferator-activated receptor gamma, *Pparγ*, in BAT was significantly lower in *db*/*db* mice than that in WT mice at 2 weeks of age but was similar at 8 weeks of age.

## 4. Discussion

The present study revealed the distinct roles of Lepr in FI and BT regulation in pups and adults using an established mouse line carrying *db* (a mutation in the gene encoding the leptin receptor long form) without *misty* (a mutation in the gene encoding Dock7, related to neural development). Regarding the main clinical manifestations such as BW, milk and food intake, and BT, although there may be a slight difference in degree, mice of both sexes displayed a similar tendency of the manifestations. Therefore, further examinations (organ weight, blood chemistry, and gene expression) were performed in males only.

A method using mismatched primers for *db* genotyping was reported [28, 29]. A novel method for *db* gene was used to establish a pure *db* mouse line. This system using a novel restriction enzyme that recognizes the mutated sequence might be easier and more reliable than previous systems (Supplementary Figure 1(a)). This method worked well, leading to the generation of a *db* mouse line lacking the *misty* mutation. The length between the *Lepr* and *Dock7* genes is 1.36 cM (chromosome: 4; NC_000070.6; Gene ID 16847 and ID 67299 are designated by NCBI, respectively). Therefore, recombination of the genes is likely in more than 1 out of 50 births [31]. As expected by natural recombination, we ultimately obtained *M db/M+*, and then a mouse line carrying *db* without the *Dock7* mutation was established (Table 1). In addition, littermates from the line were genotyped as homozygous *db* and homozygous wild-type *Lepr* 1 week after birth. BW changes and FI were examined (Figure 1 and Table 2). *db*/*db* mice displayed a higher BW after the weaning period but not in pups. Correspondingly, adult, but not pup, *db*/*db* mice displayed higher FI. The BW was not significantly different between WT and *db/db* mice at 2 weeks of age, and the brain, liver, and WAT weights were similar in *db/db* mice and WT mice. These data suggest that there is a similar role for Lepr signaling between BW regulation and adiposity regulation.

In contrast, *db*/*db* mice had lower BT in pups and adults. The results were consistent with those in Zucker rats with a leptin receptor mutation. Zucker rats displayed a lower core BT at 1 week after birth and increased FI later in life [32]. *ob* mice with leptin deficiency displayed thermogenic defects and similar milk intake, compared with control mice, at around 2 weeks of age, the preobese phase [15, 18].

The findings of the present study regarding FI and BT regulation were corroborated by the results on gene expressions of the hypothalamus and BAT. These findings were consistent with the expression of orexigenic and anorexigenic peptide genes in the hypothalamus and *Ucp1* in BAT (Figure 2). In pups, milk intake regulation may not be involved by leptin action because orexigenic and anorexigenic hypothalamic neuropeptide gene expression was not altered in WT and *db/db* mice. This finding was consistent with reports based on leptin administration experiments including an *ob*/*ob* mouse study [20, 33]. Because exogenous leptin administration and the leptin-deficient state displayed no alterations in orexigenic and anorexigenic peptide gene expression in the hypothalamus, leptin signaling, including Lepr, may not regulate FI in pups [20, 33]. After weaning, at 8 weeks of age, orexigenic and anorexigenic peptide genes in the hypothalamus may be altered, in association with higher BW and increased FI in the present study (Figures 1 and 2). The lack of leptin signaling in pups may be explained by the suppression of *Lepr* gene expression. Alternatively, the leptin signaling cascade from Lepr may be blunted. The mechanisms underlying this regulation such as the methylation state of gene promoters involved in leptin signaling should be examined [34–36].

In contrast, BT was lower in pup and adult *db*/*db* mice with long form Lepr deficiency, suggesting that Lepr, particularly the long form, is consistently involved in BT regulation. In adult mice, POMC is important for BT regulation by mediating leptin action [1, 3, 37]. POMC produces melanocyte-stimulating hormone, leading to activation of the melanocortin-4 receptor. Consequently, sympathetic tone is activated. However, in infants, in contrast to adults, *Pomc* expression levels were similar in *db*/*db* mice, suggesting that the decrease in BT is not explained by the reduced signaling of melanocortin-4 receptor involved in POMC (Figure 2(a4)). Reduced BT in *db*/*db* mice indicates that Lepr is involved in BT regulation in association with the lower expression of *UCP1* at both age, but the precise underlying mechanism has not been elucidated (Figure 2(b1)). This gene expression result may be accordant with BAT weight result of WT and *db/db* mice, where there was a significant difference between the genotypes under less significant interaction of age and genotype (Table 2). Postnatal *db/db* mice had a significantly lower expression of *PPARγ* (Figure 2(b2)). This finding indicates that decreased BT in *db/db* mice may underlie the different mechanism between pups and adults [37, 38]. For example, instead of central regulation through sympathetic tone, Lepr expressed in BAT may be involved in heat production through expression of the *Ucp1* and *Pparγ* genes [39, 40]. In addition, BT is maintained by thyroid function [41]. In the present study, thyroid function was examined. *db*/*db* mice displayed lower T4 but similar TSH levels in both ages, suggesting that central hypothyroidism is involved in the reduced BT.

The leptin surge or increased leptin in the postnatal rodents was reported, but its physiological importance remains unclear [18, 19]. The postnatal surge in leptin may set in motion a number of developmental events which do not materialize until later. The increased leptin may be involved in the maintenance of BT in pups through Lepr, partly, associated with thyroid function. Although the mechanism underlying reduced BAT *Ucp1* expression in postnatal *db*/*db* mice is unclear, it may be related to the lower BT in these mice. Based on our results, the leptin surge may be more involved in BT regulation than in FI regulation.

To the best of our knowledge, the present study is the first to compare *db/db* and *+/+* mice with the BKS background without *misty*. In the other studies using the *db/db* mouse line with the BKS background, which is relatively prone to diabetes [21], *m/m* or *m/+* mice were used as controls [42, 43]. The *misty* mutation may influence BW and findings related to sympathetic nerve activity. To compare the results from the present study, especially, in adult mice with that of other studies with mice harboring the *misty* mutation, we should consider the results from the control mice, which may be influenced by *misty*.

In conclusion, the age-dependent phenotypes were corroborated by the results of gene expressions of the hypothalamus and BAT, which have not been fully elucidated in *fa* rats and *ob* mice, especially in the preobese phase corresponding to 2 weeks of age. Using a *db* mouse line without the *misty* mutation, the different appearances of FI and BT at different ages were presented at the molecular levels, suggesting the age-dependent involvements of the leptin receptor long form in FI and BT.

## Figures and Tables

**Table 1:** DNA diagnosis of mice purchased from companies.

	Company	
	A	B
Phenotype		
Coat color/body appearance	Black/lean	Black/lean
Expected genotype		
Gene 1/gene 2	*M db*/*m+*	*M db*/*m+*
Experimental results		
*M db*/*m+*, *M+/m db*	10 (83.3)	19 (95.0)
*M db*/*M+*	2 (16.7)	1 (5.0)
*M+*/*m db*	0 (0)	0 (0)
*M+*/*m+*	0 (0)	0 (0)
*M+*/*M+*	0 (0)	0 (0)

Genes 1 and 2 are shown as a pair of genotypes of dedicator of cytokinesis 7 (*Dock7*) gene and leptin receptor (*Lepr*) gene. *M*: wild type of *Dock7*; *m*: a mutation type (*misty*) of *Dock7*; *+*: wild type of *Lepr*; *db*: a mutation type of *Lepr*. The number of mice diagnosed is described. The mice examined from the company A and company B were 12 and 20, respectively. The number in parentheses denotes the percentage.

**Table 2:** Organ weight and biochemical parameters including hormones at 2 and 8 weeks.

		2 weeks		8 weeks		*P* values (two-way ANOVA)		
Male		WT (*N* = 8)	*db*/*db* (*N* = 8)	WT (*N* = 8)	*db*/*db* (*N* = 8)	Age	Genotype	Interaction
Body weight	g	6.15 ± 0.18^a^	6.41 ± 0.21^a^	24.91 ± 0.30^b^	41.82 ± 0.75^c^	(<0.01)	(<0.01)	**<0.01**
Milk intake^#^	g/h	0.146 ± 0.015	0.153 ± 0.024^NS^					
Food intake	g/day			3.42 ± 0.09	7.60 ± 0.33^∗∗^			
Organs								
Brain	mg	355.0 ± 5.6^ab^	335.5 ± 5.9^a^	421.7 ± 3.6^c^	363.3 ± 5.1^b^	(<0.01)	(<0.01)	**<0.01**
Heart	mg	34.8 ± 2.0	30.0 ± 1.1	120.9 ± 1.5	117.9 ± 2.8	<0.01	0.055	0.660
Liver	g	0.19 ± 0.01^a^	0.19 ± 0.01^a^	1.36 ± 0.03^b^	2.79 ± 0.11^c^	(<0.01)	(<0.01)	**<0.01**
WAT	g	0.013 ± 0.002^a^	0.026 ± 0.003^a^	0.250 ± 0.013^b^	1.781 ± 0.073^c^	(<0.01)	(<0.01)	**<0.01**
BAT	mg	61.0 ± 6.5	76.5 ± 6.3	103.2 ± 8.0	198.9 ± 49.4	<0.01	0.037	0.126
Plasma								
Glucose	g/l	1.12 ± 0.05^a^	1.01 ± 0.04^a^	2.29 ± 0.19^a^	6.14 ± 0.89^b^	(<0.01)	(<0.01)	**<0.01**
TG	g/l	0.68 ± 0.10	0.79 ± 0.12	1.04 ± 0.13	1.20 ± 0.17	<0.01	0.284	0.844
Insulin	ng/ml	0.40 ± 0.03^a^	0.59 ± 0.08^a^	0.99 ± 0.09^a^	9.61 ± 3.53^b^	(<0.01)	(<0.019)	**0.024**
Leptin	ng/ml	4.0 ± 1.1^a^	46.6 ± 5.4^b^	1.1 ± 0.3^a^	94.6 ± 7.2^c^	(<0.01)	(<0.01)	**<0.01**
Corticosterone	ng/ml	43.6 ± 7.1^a^	56.3 ± 8.7^a^	148.0 ± 33.2^a^	292.3 ± 52.1^b^	(<0.01)	(<0.013)	**0.034**
T3	ng/ml	2.80 ± 0.01	2.92 ± 0.14	1.16 ± 0.03	1.22 ± 0.04	<0.01	0.235	0.688
T4	ng/ml	98.6 ± 3.5	81.5 ± 5.2	41.6 ± 1.5	33.7 ± 0.9	<0.01	<0.01	0.172
TSH	*μ*IU/ml	6.71 ± 0.47	7.06 ± 0.40	0.99 ± 0.23	1.85 ± 0.75	<0.01	0.236	0.617

Female		WT (*N* = 5)	*db*/*db* (*N* = 5)	WT (*N* = 5)	*db*/*db* (*N* = 5)	Age	Genotype	Interaction
Body weight	g	6.01 ± 0.11^a^	6.37 ± 0.14^a^	19.38 ± 0.21^b^	39.27 ± 0.92^c^	(<0.01)	(<0.01)	**<0.01**
Milk intake	g/h	0.093 ± 0.024	0.102 ± 0.031^NS^					
Food intake	g/day			3.03 ± 0.09	7.34 ± 0.43^∗∗^			

WT: wild type (homozygous wild type in leptin receptor gene); *db*/*db:* leptin receptor deficiency (homozygous mutants in leptin receptor gene); BW: body weight; WAT: white adipose tissue; BAT: brown adipose tissue; TG: triglyceride; T3: triiodothyronine; T4: thyroxine; TSH: thyroid-stimulating hormone. ^#^The number of male samples for milk intake measurement was 7. All data were obtained from mice after 3 h fasting except milk and food intake (described in detail in Materials and Methods). Values are the mean ± SE. Values except milk and food intake were analyzed by two-way ANOVA (interaction, age, and genotype) followed by the Tukey-Kramer method when significant. Bold values mean the significance in the interaction. In the case, the main effect of age and genotype is shown by the parenthesis as a reference. Values of milk and food intake were analyzed by unpaired Student's *t*-test, respectively. The same superscript letter and NS indicate that the difference was not significant. ^∗∗^ *P* < 0.01 vs. WT mice at the respective age.

**Table 3:** Core body temperature in light and dark phases at 2 and 8 weeks.

		2 weeks		8 weeks		*P* values (two-way ANOVA)		
Male		WT	*db/db*	WT	*db/db*	Age	Genotype	Interaction
Time measured (h)		*N* = 7	*N* = 6	*N* = 5	*N* = 5			
8:00	°C	35.70 ± 0.22	33.93 ± 0.20	36.66 ± 0.21	34.51 ± 0.34	<0.01	<0.01	0.455
20:00	°C	36.38 ± 0.18^NS^	34.99 ± 0.21^∗^	38.10 ± 0.28^∗^	36.60 ± 0.15^∗∗^	<0.01	<0.01	0.795

Female		WT	*db/db*	WT	*db/db*	Age	Genotype	Interaction
Time measured (h)		*N* = 5	*N* = 5	*N* = 5	*N* = 5			
8:00	°C	36.14 ± 0.09	33.89 ± 0.15	36.64 ± 0.13	33.96 ± 0.13	0.051	<0.01	0.126
20:00	°C	37.36 ± 0.11^∗∗^	34.96 ± 0.15^∗∗^	37.90 ± 0.10^∗∗^	35.89 ± 0.28^∗∗^	<0.01	<0.01	0.279

WT: wild type (homozygous wild type in *Lepr* gene); *db*/*db*: leptin receptor deficiency (homozygous mutants in *Lepr* gene). Values are the mean ± SE. Values were analyzed by two-way ANOVA (interaction, age, and genotype). Values of the respective genotype male or female mice at the same age were analyzed by paired Student's *t*-test. NS: not significant; ^∗^ *P* < 0.05, ^∗∗^ *P* < 0.01 vs. male or female mice of the respective genotype at the same age at 8:00 h.

## Data Availability

The data used to support the findings of this study are available from the corresponding author upon request.

## Supplementary Materials

Supplementary Materials Supplementary data include sequences of primer for genetic diagnosis and real-time PCR as a table and genotyping of leptin receptor (Lepr) and dedicator of cytokinesis 7 (Dock7) genes related to *db* and *misty* as a figure.
