# Supplementary material for: Food Intake and Core Body Temperature of Pups and Adults in a db Mouse Line Deficient in the Long Form of the Leptin Receptor without Misty Mutation
Source: J Diabetes Res. 2018 Dec 3;2018:9670871. doi: 10.1155/2018/9670871 (PMC6304817; doi:10.1155/2018/9670871)
Supplement: Supplementary Materials — Supplementary data include sequences of primer for genetic diagnosis and real-time PCR as a table and genotyping of leptin receptor (Lepr) and dedicator of cytokinesis 7 (Dock7) genes related to db and misty as a figure. [file 9670871.f1.pdf]

Supplementary Figure 1:

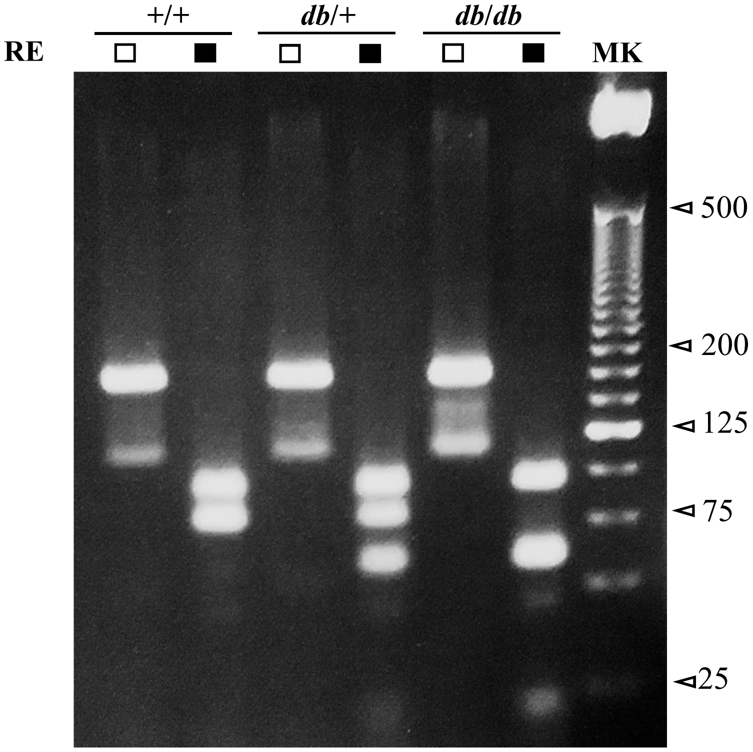

(a)

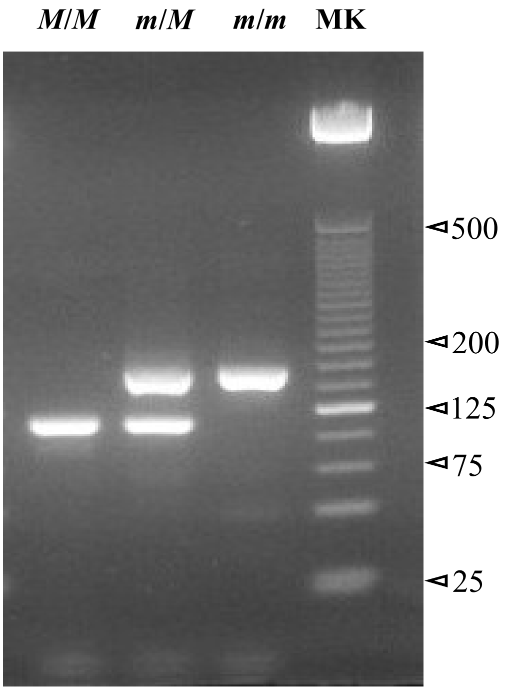

(b)

Supplementary Table 1: Primer sequences for genetic diagnosis and real-time PCR.

Gene	Accession number	Sequence
Genes trageted for genetic diagnosis		
Lepr	NC_000070	F: 5'-AGAACGGACACTCTTTGAAG-3'
		R: 5'-TCTAATAAGCTCATCAAATGTTA-3'
Dock7	NC_000070	F: 5'-CTGTCCACATAATGATTTCAATA-3'
		R1: 5'-CTGGCAGGCAGAACTGCC-3'
	FJ590430	R2: 5'-CAATCGCAAACGCATTAGTAAA-3'
Genes targeted for real-time PCR		
Gapdh	NM_008084	F: 5'-CCGCATCTTCTTGTGCAGTGCC-3'
		R: 5'-GGGGTCGTTGATGGCAACAATCTC-3'
Agrp	NM_007427	F: 5'-GAGTTCCCAGGTCTAAGTCTGAATG-3'
		R: 5'-ATCTAGCACCTCCGCCAAAG-3'
Cart	NM_013732	F: 5'-ACGAGAAGAAGTACGGCCAA-3'
		R: 5'-CCCGATCCTGGCCCCTTT-3'
Npy	NM_023456	F: 5'-TCATCACCAGACAGAGATATG-3'
		R: 5'-GTGCTTTCCTTCATTAAGAGG-3'
Pomc	NM_008895	F: 5'-CTGCTTCAGACCTCCATAGA-3'
		R: 5'-GGATGCAAGCCAGCAGGTT-3'
Ucp1	NM_009463	F: 5'-TACCAAGCTGTGCGATGTCCA-3'
		R: 5'-GCACACAAACATGATGACGTTCC-3'
Pparγ	NM_011146	F: 5'-TGTGGGGATAAAGCATCAGG-3'
		R: 5'-GGCAGTTAAGATCACACCTAT-3'

Lepr : leptin receptor; *Dock7* : dedicator of cytokinesis 7;

Gapdh : glyceraldehyde-3-phosphate dehydrogenase; *Agrp* : agouti related neuropeptide;

Cart : cocaine- and amphetamine- regulated transcript; *Npy* : neuropeptide Y;

Pomc : pro-opiomelanocortin; *Ucp* : uncoupling protein;

Ppar : peroxisome proliferator activated receptor.

F: forward primer; R: reverse primer.

Dock7 ; wild type is amplified by R1; a mutant gene (*misty*) is amplified by R2.

The two genes are amplified by the same forward primer.
